# The Effect of Prior Upper Body Exercise on Subsequent Wingate Performance

**DOI:** 10.1155/2014/329328

**Published:** 2014-05-07

**Authors:** Marie Clare Grant, Robert Robergs, Marianne Findlay Baird, Julien S. Baker

**Affiliations:** ^1^Institute of Clinical Exercise and Health Science, Exercise Science Research Laboratory, School of Science, Faculty of Science and Technology, University of the West of Scotland, Hamilton ML3 OJB, UK; ^2^Division of Sport and Exercise Sciences, School of Social & Health Sciences, Abertay University, Bell Street, Dundee DD1 1HG, UK; ^3^School of Human Movement Studies, Charles Sturt University, Bathurst, NSW 2795, Australia

## Abstract

It has been reported previously that the upper body musculature is continually active during high intensity cycle ergometry. The aim of this study was to examine the effects of prior upper body exercise on subsequent Wingate (WAnT) performance. Eleven recreationally active males (20.8 ± 2.2 yrs; 77.7 ± 12.0 kg; 1.79 ± 0.04 m) completed two trials in a randomised order. In one trial participants completed 2 × 30 s WAnT tests (WAnT1 and WAnT2) with a 6 min recovery period; in the other trial, this protocol was preceded with 4 sets of biceps curls to induce localised arm fatigue. Prior upper body exercise was found to have a statistically significant detrimental effect on peak power output (PPO) during WAnT1 (*P* < 0.05) but no effect was observed for mean power output (MPO) (*P* > 0.05). Handgrip (HG) strength was also found to be significantly lower following the upper body exercise. These results demonstrate that the upper body is meaningfully involved in the generation of leg power during intense cycling.

## 1. Introduction

High intensity cycle ergometry has been widely employed in sport and exercise science research to assess indices of muscular performance [1, 2]. Among these power variables, the measurement of PPO has received considerable interest. PPO measurement has traditionally been attributed to the activity of the lower body musculature. Previous work and recent investigations in our laboratory have shown that the upper body may significantly contribute to PPO [2–4]. Surface electromyography (sEMG) has revealed that several upper body muscles (brachioradialis (BR), biceps brachii (BB), triceps brachii (TB), and upper trapezius (UT)) are continually active during high intensity cycle ergometry when a standard handlebar grip is used [4]. With the current cycle ergometer design, evidence suggests that the forearm muscles and therefore the handlebar grip are influential to overcome high resistive loads to produce an optimum PPO. This is supported by the findings of Baker et al. (2001) [2] who found PPO to be significantly greater when a standard handlebar grip was in place compared to no grip (*P* < 0.05).

The effects of prior upper body exercise on subsequent cycling performance have previously been examined [5]. In this study, blood lactate concentrations [La^−^] were elevated, via arm-crank exercise, and dynamic performance during two 30 s WAnT was assessed. It was found that prior arm exercise was related to a decline in PPO during the second WAnT with the authors suggesting that the resulting elevated [La^−^] caused an increased uptake in La^−^ and H^+^ by the inactive leg muscles, leading to an overall performance decrement. Karlsson et al. (1975) [6] have also suggested that a period of exhausting anaerobic exercise by the arms or legs might decrease the performance time of anaerobic effort in the nonexercising arm or leg region due to the possible detrimental effects of elevated [La^−^] and [H^+^].

Although it is now known that La^−^ *per se* does not directly cause muscle fatigue, a rise in other metabolic by-products such as inorganic phosphate (P_i_) [7], P_i_ is likely to play a major role in muscular fatigue during high intensity exercise. Potential mechanisms whereby high [P_i_] can impair contractile function thus affecting muscle force production include hindering crossbridge transition to the strongly bound high force state; reducing myofibrillar calcium (Ca^2+^) sensitivity; increasing the opening probability of the sarcoplasmic reticulum (SR) Ca^2+^ release channels; inhibiting Ca^2+^ uptake by the SR; and precipitating with the Ca^2+^ in the SR, so decreasing the amount of Ca^2+^ available for release [8]. A rise in metabolic by-products is concomitant with partial depletion and inhibition of the phosphagen and glycolytic energy systems [9] during exercise may affect muscle function in the nonexercising arm or leg [10]. This effect may contribute to decline in muscular force production and overall performance. Based on previous research highlighting the importance of the upper body musculature in high intensity cycle ergometer performance, it is plausible that when the upper body is fatigued, it will be less able to support or stabilize the body to allow for more effective leg power development [1].

The experimental design of the present study was largely based on that of Bogdanis et al. [5] with the aim of further examining the effects of prior fatiguing upper body exercise on subsequent WAnT performance. A secondary aim was to investigate if HG strength was correlated with power profiles.

## 2. Methodology

### 2.1. Participants

Eleven healthy, recreationally active males (20.8 ± 2.2 yrs; 77.7 ± 12.0 kg; 1.79 ± 0.04 m) volunteered to participate in the study. The study was approved by the university ethical committee and all participants completed an informed consent form and medical history questionnaire. Participants were instructed to maintain their normal diet during the days leading up to and on the days of testing and they were asked to refrain from vigorous exercise and avoid the consumption of caffeine and alcohol during the 24 hours preceding the testing date. Food was not consumed during testing and water was available* ad libitum*.

Participants attended the laboratory on three separate occasions, at the same time of day, separated by 48 to 72 hrs. Participants did not report any muscle soreness before any of the sessions. The first session was a familiarisation session to control for the potential effects of learning a novel task and increase reliability of the results. During this session participants were briefed on experimental procedures, instructed, and familiarised with high intensity cycle ergometry, bicep curls, and maximal HG testing. Body mass (kg), stature (m), and 1RM were also determined during this session.

**Figure 1:**
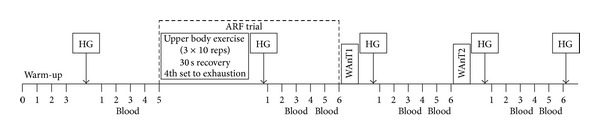
Schematic representation of both experimental protocols. HG = hand grip; WAnT1 = Wingate Test 1; WAnT2 = Wingate Test 2; and BS = blood fingertip sample.

The following two experimental trials were completed in a randomised order. For the no arm fatigue (NOF) trial, participants were required to perform two maximal 30 s sprints (WAnT1 and WAnT2) on a cycle ergometer with a standard handlebar grip, separated by 6 min passive recovery. In the other arm fatigue (ARF) trial, bicep curls were completed prior to WAnT1 and WAnT2. Blood [La^−^] and handgrip strength were obtained at predetermined time points throughout the protocols (Figure 1).

### 2.2. Cycle Ergometry

A leg cycle ergometer (Monark 894E, Vansbro, Sweden) was used for each experimental protocol. For each participant the saddle height was adjusted so their knee remained slightly flexed after the completion of the power stroke (with final knee angle approximately 170–175°). Toe clips were used to ensure that the participants' feet were held firmly in place and in contact with the pedals. The cycle ergometer was connected to a PC to allow for data capture via the Monark anaerobic test software (version 2.24.2).

Before any experimental testing, each individual completed a standardised warm-up on the cycle ergometer (3 min at 60 rpm, 2 kg resistance).

For both WAnT1 and WAnT2, participants were given a rolling start before resistive force application. Once the subjects had accelerated to 60 rpm the weight basket automatically dropped and participants began to pedal maximally. Each participant was required to pedal with maximum effort for a period of 30 s against a fixed resistive load of 75 grams per kilogram (g·kg^−1^) total body mass as recommended by Bar-Or (1987) [11]. All participants were given the same level of verbal encouragement and instructed to remain seated for the duration of the test while maintaining a standard handlebar grip. Variables obtained from the Monark anaerobic test software (version 2.24.2) were PPO (W), relative PPO (W·kg^−1^), MPO (W), and relative MPO (W·kg^−1^). For the population used within the study the WAnT has a high test-retest reliability (*r* = 0.95–0.97) [11].

### 2.3. Bicep Curls

In a familiarisation session before any experimental testing, each individual carried out a series of bicep curls to allow for their 1RM to be estimated. With each set the weight was adjusted so that no more than 10 repetitions could be completed with the final weight. Participants were given 2-minute recovery between each set. All participants were familiar with the exercise; therefore no more than three sets were required. The Brzycki formula was then used to estimate 1RM based on the final weight and repetitions recorded (1) [12]. 70% of the 1RM was subsequently calculated for each participant to establish the weight required to fatigue the arms for protocol 2:

(1) $$\begin{array}{c} 1\text{RM}=\frac{\left(\text{weight}\;\text{lifted}\right)}{\left[1.0278-\left(\text{repetitions}\times 0.0278\right)\right]}. \end{array}$$

For protocol 2 (ARF), participants completed 3 sets of 10 repetitions and a 4th set until exhaustion (R30 s between sets) at 70% 1RM. During the bicep curls, participants' palms were in the supinated position and they were instructed to keep the feet a shoulder width apart with their elbows close to their sides and complete each curl with a continuous, smooth movement with minimum body disruption.

### 2.4. Handgrip

HG strength (kg) was measured 1 min after warm-up and 1 min after each exercise bout (Figure 1). Each maximal static HG test was completed with the participant's dominant hand, while being in a seated position using a HD dynamometer (Model TKK5001, Takei, Japan).

#### 2.4.1. Blood Sampling

Capillary blood samples (30–50 *μ*L) for the measurement of blood [La^−^] were taken from the fingertip using standard lancets and capillary tubes. Samples were taken at rest, 3 min following warm-up and 3 and 5 min following each exercise bout (Figure 1). All samples were immediately mixed (2 min) and duplicate samples were analysed to determine the whole blood lactate concentration (Analox P-LM5, Analox Instruments Ltd, London, UK).

The full protocol for each testing session is outlined in Figure 1.

#### 2.4.2. Statistical Analysis

Data was statistically analysed using SPSS (version 20) (IBM, Armonk, NY, USA). For a single missing data point, data was replaced with a mean difference adjusted value for the individual compared to the other trial data point. For each of peak power, relative peak power, mean power, relative mean power, and fatigue index (FI, %), repeated measures two-way (2 [TRIAL] × 2 [TEST]) ANOVAs were performed to detect main effect and interaction effects. For the blood lactate data, a balanced design was only evident for the postexercise data (3 and 5 min following exercise). For these data, data were analyzed by repeated measures three-way (2 [TRIAL] × 2 [TEST] × 2 [TIME]) ANOVA. For HG data, a balanced design was evident when using the post-warm-up data for the NOF trial and the post-arm fatigue test data for the ARF trial as the preexercise data. Preexercise was then compared to postexercise (1 min WAnT1 versus 1 min WAnT2) using a repeated measures two-way (2 [TRIAL] × 3 [TIME]) ANOVA. For all data variables, specific contrasts were performed to test for mean differences for significant main or contrast effects. Isolated paired mean differences outside of the balanced ANOVA designs were assessed by a paired *t*-test. Pearson's correlation analysis was used to determine the correlation between PPO and HG strength. Effect size statistics (ES) for selected statistically significant *t*- and *F*-ratios were also established. These calculations were based on Cohen's (*d*) classification of a small (0.2 ≤ *d* < 0.5), moderate (0.5 < *d* < 0.8), and large (*d* ≥ 0.8) ES [13]. Significance was set* a priori *at *P* < 0.05. All data is presented as mean ± standard deviation (SD).

## 3. Results

### 3.1. Cycle Parameters

There was a significant main effect for the TEST for each of PP0 (W: *F*_1,8_ = 19.5, *P* < 0.01, *r*_df_ = 0.84), relative PP0 (W·kg^−1^: *F*_1,8_ = 22.6, *P* < 0.01, *r*_df_ = 0.86), and relative MPO (*F*_1,8_ = 43.8  *P* < 0.01, *r*_df_ = 0.91) (Table 1), revealing lower power values for WAnT2 compared to WAnT1. Peak power produced in WAnT1 was lower in the ARF protocol compared to NOF protocol (*P* < 0.01, *r*_df_ = 0.75); however, no significant interaction was found (*P* > 0.05). No significant differences were found in FI (%) for TEST or TRIAL (*P* > 0.05) (NOF: 57.0 ± 10.5% and 57.8 ± 10.7% for WAnT1 and WAnT2, respectively, ARF: 57.3 ± 9.9% and 59.1 ± 9.6% for WAnT1 and WAnT2).

### 3.2. Blood Lactate

**Figure 2:**
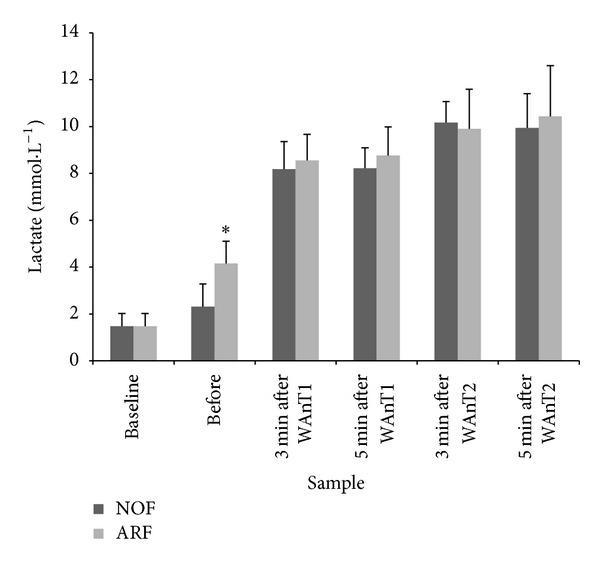
Blood [La^−^] responses prior to and during recovery from each of WAnT1 and WAnT2.  ∗  =  significant difference between NOF and ARF.

The blood lactate response to each of the experimental protocols is displayed in Figure 2. There was a significant TIME effect (*F*_1,8_ = 15.8, *P* < 0.01, *r*_df_ = 0.81) and a significant TRIAL × TEST interaction (*P* = 0.041, *r*_df_ = 0.57). The three-way interaction effect of TRIAL × TIME × TEST revealed a trend toward statistical significance (*P* = 0.089, *r*_df_ = 0.54). Blood lactate was significantly higher 5 min after the arm fatigue exercise in the ARF trial compared to post-warm-up in the NOF trial (*P* = 0.001). Based on the main effect and interaction ANOVA results, blood lactate was significantly higher throughout the recovery after WAnT2 than WAnT1. In addition, the interaction effect was caused by a net decrease in blood lactate between 3 and 5 min of recovery in WAnT2, whereas blood lactate continued to increase between 3 and 5 min of recovery after WAnT1.

### 3.3. Handgrip

**Figure 3:**
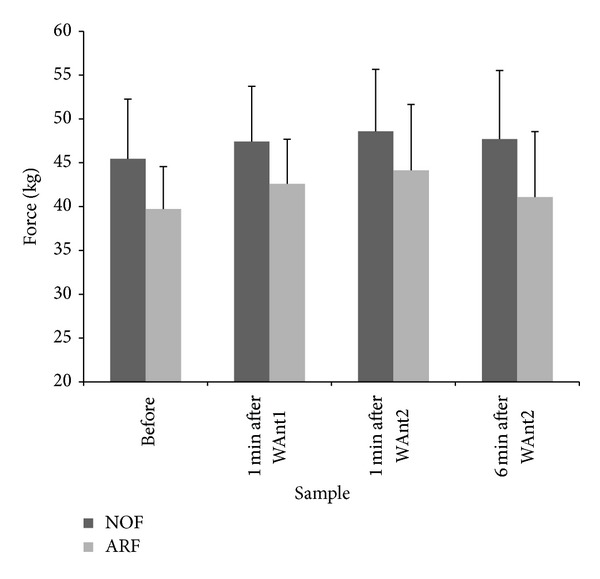
Handgrip strength (kg) measured before and after exercise for both trials.

The HG strength response to each of the experimental protocols is displayed in Figure 3. There was a significant TRIAL effect (*P* = 0.004,  *r*_df_ = 0.81). Therefore, HG strength was significantly lower at all time points for the ARF versus NOF trial.

**Figure 4:**
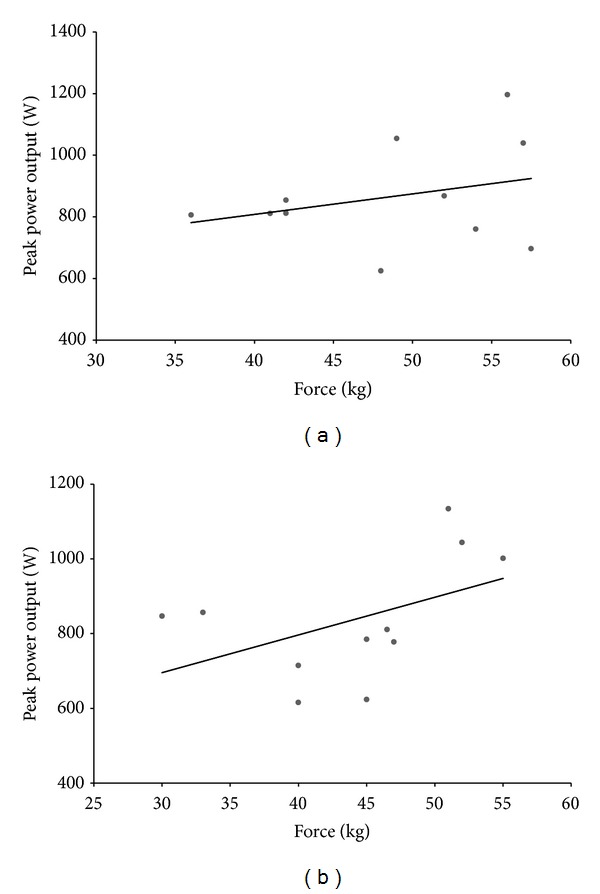
Correlations between handgrip strength after WAnT2 and peak power from WAnT2 for (a) NOF (*r* = 0.29,  *P* = 0.38) and (b) ARF (*r* = 0.59,  *P* = 0.06).

There was a nonsignificant correlation between HG strength and PPO during NOF (*r* = 0.29,  *P* = 0.38). However, in the ARF where bicep curls were completed before WAnT1, there was a meaningful trend of a positive linear relationship (*r* = 0.59,  *P* = 0.06) between PPO from WAnT2 and HG strength after WAnT2 (Figure 4).

## 4. Discussion 

The main aim of the present study was to evaluate the effects of prior fatiguing upper body exercise on subsequent high intensity cycle ergometer performance. The results demonstrate that fatiguing the upper body had a detrimental effect on PPO during WAnT1 with nonsignificant impact on any other power variables. Interestingly, the connection between prior upper body exercise and PPO was best revealed as a fair correlation between HG strength and PPO in protocol 2 (Figure 4). Consequently, the functional connection between HG strength and PPO is more relevant after prior exercise of the upper body.

In the ARF protocol, HG strength was lower at all measured time points compared to the NOF protocol. Furthermore, PPO was significantly lower (*P* < 0.05) in WAnT1 in the ARF protocol compared to the NOF protocol which suggests that in the absence of leg fatigue, the strength of the grip upon the handlebar may be influencing PPO. This is in agreement with the findings of Baker and colleagues (2001) [1] who found that handlebar grip was essential in the production of PPO. Perhaps unexpectedly, the only correlation between PPO and HG strength reaching significance was between PPO obtained in WAnT2 and HG strength after WAnT2 in the ARF protocol (*r* = 0.59,  *P* = 0.06). A possible explanation for this finding is that as HG strength shows signs of recovery following the fatiguing arm exercise (Figure 3), the relationship between HG strength and PPO becomes more evident.

The lack of statistically significant differences between the two protocols in the MPO data is likely to be partly due to the added variability of this measure compared to PPO. However, it can be speculated that the prior high intensity upper body exercise would have resulted in faster $\dot{\text{V}}{\text{O}}_{2}$ kinetics facilitating an earlier and greater shift to aerobic metabolism in the first sprint in protocol 2 (ARF). This shift has the potential to improve MPO by reducing the O_2_ deficit and rate of fatigue induction [14, 15]. This reduction in fatigue can be highlighted through the lack of difference in FI (%) found between WAnT1 and WAnT2 in both protocols.

Despite the statistically significant increase in blood [La^−^] during high intensity exercise, it is now widely accepted that La^−^ does not have a direct role in muscular fatigue [16]. However, the rate of blood [La^−^] accumulation and removal can be used as a measure of the status of muscle metabolism with trained individuals reported as having a greater lactate transport capacity than their untrained counterparts [17]. During intense exercise, the predominate mechanism which moves La^−^ and H^+^ out of contracting muscle is the monocarboxylate transporter system, MCT1 and MCT4 [18, 19], with the transport efficiency dependent upon various factors including intramuscular and blood pH, density of MCT1 and MCT4, and on blood flow in working muscles and other tissues [20]. In the present study, there was a small increase in the standard deviation values from 3 to 5 min after exercise suggesting participant variability in lactate transport efficiency which reflects possible between-subject differences in training status.

During ARF, participants commenced the WAnTs with significantly greater circulating blood [La^−^]. Apart from the added intense upper body exercise that induced this increase, it has also been established that the upper body has a higher percentage of type II fibres than the lower body which in turn causes upper body musculature to be less efficient in lactate clearance and subsequently also has a slower recovery [21, 22]. Despite the metabolic benefits of La^−^ production now being widely recognised, there is a definite association between elevated blood [La^−^] and impaired exercise performance [16]. Therefore, this elevated [La^−^] in the ARF protocol is likely to be a reflection of other metabolic disturbances including metabolic acidosis and an increase in intramuscular P_i_ and blood K^+^ thus partially accounting for the decrease in PPO [7, 23]. It is important to highlight that despite the common belief that muscle acidosis is a major cause of fatigue, there is now reasonable evidence to suggest that the effects of H^+^ on force production may be largely temperature dependent and may have little direct effect on human muscle at physiological temperature [24].

In addition to the metabolic disturbances within the muscles, prior high intensity exercise also causes partial depletion and inhibition of the phosphagen and glycolytic energy systems leading to decline in muscular force production. In terms of energy depletion influencing subsequent performance, PCr recovery is likely to be a key factor. Research has shown that after exhaustive exercise, near complete replenishment of PCr may take from <5 min to in excess of 15 min, depending on the extent of PCr depletion, severity of metabolic acidosis (slower if more acidic), and the muscle motor unit and fibre type characteristics of the exercised muscle [7]. Therefore, in the present study it is unlikely that the 6-minute rest period between exercise bouts would be sufficient for complete replenishment of PCr stores, thus having a detrimental effect on power profiles obtained following a previous bout of high intensity exercise. Furthermore as outlined previously, blood [La^−^] remains elevated, suggesting intramuscular H^+^ activity also remains higher, thus slowing the replenishment process. This is supported by Bogdanis and colleagues [25] who found that decreased [PCr] did result in a reduction in power output. However, it is likely that the detrimental effects of prior exercise on subsequent performance will be muted when the recovery period is adequate [26, 27]. For example, when Bouhlel et al. [10] investigated the possible impact on estimated peak anaerobic power when a leg test was preceded by an arm test (or vice versa), they found that subsequent performance was not reduced. The 8 min recovery period within this study suggests that the opposite muscle group is unaffected by any continuing metabolic disturbances or other changes from the preceding bout of exercise if the recovery period is adequate.

In a previous study by Bogdanis and colleagues [5], a very similar methodology was used. The authors aimed to elevate [La^−^] through prior arm exercise (arm ergometry) and determine the effects of this on subsequent high intensity cycle ergometry performance. In contrast to our results they found PPO to be significantly lower during the second sprint following prior arm exercise but similarly there were nonsignificant changes in MPO between the two protocols. It is difficult to directly compare results with Bogdanis et al. [5] due to differences in equipment, participants, and experimental procedures employed. We have hypothesised that the differences between the two investigations may be due to our upper body exercise (bicep curls) having a directly fatiguing effect on HG strength which we hypothesised would be more likely to affect PPO during the initial sprint. We interpret our results to confirm this hypothesis.

## 5. Limitations

As with most maximal cycle ergometer tests, prior to the load being applied there was an initial high rpm. This inertia was not accounted for in the calculation; therefore the considerable energy which had already been accumulated before the 30 s test may have resulted in a possible overcalculation of PPO and MPO [28].

All participants were physically active and accustomed to high intensity exercise. However no physiological fitness testing was undertaken prior to data collection which meant there was no control over the exercise capacity of each individual. This is an extraneous variable which may have led variation in power profiles observed among participants which was not related to the exercise protocol.

## 6. Conclusion

In conclusion, this investigation has shown that prior fatiguing upper body exercise has a statistically significant detrimental effect on PPO during the first of two WAnTs. This can be related to a number of factors, including the decrease in HG strength following the upper body exercise suggesting the upper body is less able to help overcome the high resistive loads, confirming results of previous investigations which suggest that the upper body is crucial in achieving an optimum PPO. It was also found that MPO was able to be maintained, which could be explained by prior intense exercise resulting in faster $\dot{\text{V}}{\text{O}}_{2}$ kinetics and therefore increasing the contribution from oxidative metabolism.

## Figures and Tables

**Table 1:** Power output variables recorded during WAnT1 and WAnT2 for both experimental conditions.

	WAnT1 PPO (W)	WAnT2 PPO (W)	WAnT1 PPO (W·kg^−1^)	WAnT2 PPO (W·kg^−1^)	WAnT1 MPO (W)	WAnT2 MPO (W)	WAnT1 MPO (W·kg^−1^)	WAnT2 MPO (W·kg^−1^)
NOF	980.0 ± 166.5	865.4 ± 168.1*	12.7 ± 1.5	11.2 ± 1.7*	656.0 ± 84.0	589.2 ± 89.3	8.5 ± 0.5	7.6 ± 0.7*
ARF	929.9 ± 167.7	871.7 ± 22.69*	12.0 ± 1.3	11.2 ± 2.0*	649.3 ± 86.6	576.4 ± 81.8	8.4 ± 0.7	7.5 ± 0.7*

*This indicates significant differences between WAnT1 and WAnT2 (*P* < 0.05).
